# Contrast-Enhanced Ultrasound

**DOI:** 10.1155/2015/865028

**Published:** 2015-05-28

**Authors:** Hui-Xiong Xu, Hans Peter Weskott, Ji-Bin Liu, Rong-Qin Zheng

**Affiliations:** ^1^Department of Medical Ultrasound, Shanghai Tenth People's Hospital, Tongji University School of Medicine, No. 301, Yanchangzhong Road, Shanghai 200072, China; ^2^Thyroid Institute, Tongji University School of Medicine, No. 301, Yanchangzhong Road, Shanghai 200072, China; ^3^Central Ultrasound Department, Klinikum Siloah, KRH, Roesebeckstrasse 15, 30449 Hannover, Germany; ^4^Department of Radiology, Thomas Jefferson University, 7th Floor Main Building, 132 S 10th Street Philadelphia, PA 19107, USA; ^5^Department of Ultrasound, The Third Affiliated Hospital of Sun Yat-Sen University, Guangzhou 510630, China

The recent decade has witnessed the great improvement of contrast-enhanced ultrasound (CEUS) and its extensive use in clinical practice, which is undoubtedly the major breakthrough in the field of diagnostic ultrasound in recent years. The concept of CEUS can be looked back to 60s in the last century, whereas only in 2000s CEUS regained increasing attention in both clinical practice and basic research. The current popularization of CEUS is largely due to the emergence of low acoustic power contrast-specific imaging mode and microbubble-based contrast agent filled with inert gas. After administration of ultrasound contrast agent intravenously, the low acoustic power contrast-specific imaging mode facilitates visualization of the nonlinear signals from the microbubbles in the circulation and suppresses the linear signals from the surrounding tissues, which leads to an improved signal-to-noise ratio and facilitates depiction of macro- and microcirculation of the region of interest (ROI) noninvasively. The low acoustic power also limits the damage to the microbubbles under acoustic push; thus, more microbubbles will remain in the circulation and a long time CEUS depiction is available [1].

CEUS has greatly reshaped the role of ultrasound in clinic practice. It has been demonstrated that CEUS is helpful in characterization and detection of focal lesions in various organs such as liver, gallbladder, pancreas, and kidney. In the commentary titled “Contrast-Enhanced Ultrasound (CEUS) in the Diagnosis of Hepatocellular Carcinoma and Intrahepatic Cholangiocarcinoma: Controversy over the ASSLD Guideline,” the authors proposed an interesting issue in the area of CEUS for liver lesion diagnosis that CEUS has been dropped out from the ASSLD guideline as an imaging modality for clinical diagnosis of HCC, which is one of the hot spots and arouses a lot of different opinions from scholars worldwide. One of the reasons for the drop-out is that some authors believe that CEUS is not able to make differentiation between hepatocellular carcinoma (HCC) and intrahepatic cholangiocarcinoma (ICC) [2, 3]. The commentary summarized the different opinions for the role of CUES in differentiation between HCC and ICC in recent years. It is concluded that prospective studies with strict design and large case series are mandatory to solve the controversies and stratification of ICC in terms of tumor size and liver background is also essential. In this special issue, some authors found an interesting phenomenon that post-CEUS US image can enhance the depiction of focal liver lesions in the article titled “Effects of Gray Scale Ultrasonography Immediate Post Contrast on Characterization of Focal Liver Lesions.” The underlying mechanism and possible impact to clinical practice, however, are still unknown and need more evidence for clarification.

Pancreas is another organ of interest for nonhepatic CEUS that three related articles were presented in this special issue. In the article “Utility of Contrast-Enhanced Transabdominal Ultrasonography to Diagnose Early Chronic Pancreatitis,” using the quantitative CEUS, the authors found that the ratio of blood flow in the superior mesenteric artery and pancreatic parenchyma increased with the grade of chronic pancreatitis and was significantly higher in patients with chronic pancreatitis than in patients with early chronic pancreatitis and control participants, which indicated that CEUS may be a safe and convenient method for diagnosis of early chronic pancreatitis. This attempt is interesting; however, more evidence is needed to testify the conclusion. In the article titled “Application of Contrast-Enhanced Ultrasound in Cystic Pancreatic Lesions Using a Simplified Classification Diagnostic Criterion,” the cystic lesions of the pancreas were classified into four simplified types: type I unilocular cysts; type II microcystic lesions; type III macrocystic lesions; and type IV cystic lesions with solid components or irregular thickening of the cystic wall or septa. The results of enhanced CT were considered the gold standard. Finally, CEUS has obvious superiority over US in the classification in cystic pancreatic lesions and CEUS results showed substantial agreement with CT. The results indicated that CEUS might be more accurate in the diagnosis of cystic pancreatic lesions. This study is clinically relevant since more and more cystic pancreatic lesions have been detected in clinical practice due to the wide-spread application of modern imaging techniques and its management is sometimes a dilemma. In the article of “Contrast-Enhanced Endoscopic Ultrasonography for Pancreatic Tumors,” the authors found that contrast-enhanced endoscopic ultrasonography could be useful for distinguishing pancreatic cancer from other solid pancreatic lesions and for histological differentiation of pancreatic cancer. The information provided in this article is also meaningful in clinical practice [4].

In the article of “Use of Contrast-Enhanced Ultrasound (CEUS) to Study Relationship between Serum Uric Acid and Renal Microvascular Perfusion in Diabetic Kidney Disease,” the authors found that hyperuricemia in diabetic kidney disease patients was associated with a renal ultrasound image suggestive of microvascular hyperperfusion. The CEUS parameter AUC1 holds promise as an indicator for renal microvascular hyperperfusion, while AUC2 might be a useful indicator of declining glomerular filtration rate in DKD patients with decreased excretion of uric acid. Thus, CEUS might be an imaging marker for early diagnosis of diabetic kidney disease. This result is meaningful since till now there is no imaging method that can be used to depict the hyperperfusion in early diabetic kidney diseases.

CEUS is also extensively involved in ultrasound-guided intervention procedures in that CEUS is useful to guide puncture and evaluate the treatment response. In the article titled “The Role of Contrast-Enhanced Ultrasound in Selection Indication and Improve Diagnosis for Transthoracic Biopsy in Peripheral Pulmonary Lesions,” the authors used CEUS to guide transthoracic biopsy for peripheral pulmonary lesions, which yielded a high diagnostic success rate of 96.3% versus 80% for conventional US guidance. Therefore, CEUS is useful to guide biopsy since it can avoid the areas of necrosis in the lesions and more satisfactory specimens can be expected.

Some new techniques are also tentatively used together with CEUS. In the article “Application of Combined Two-Dimensional and Three-Dimensional Transvaginal Contrast Enhanced Ultrasound in the Diagnosis of Endometrial Carcinoma,” the authors concluded that 3D-CEUS (i.e., combination of 3D ultrasound and CEUS) is a useful supplement to 2D-CEUS, which may offer direct, accurate, and comprehensive diagnosis of early endometrial carcinoma. However, the real impact of this new technique on clinical practice needs more confirmation in future studies. The inter- and intraobserver variability should also be evaluated.

Despite of the above-mentioned advances, in the level of physics, the present CEUS technology is hard to suppress all the tissue signals since the tissue can also generate nonlinear signals. On the other hand, it is found that the tissue does not generate a subharmonic response (i.e., signal at half the transmit frequency); thus, subharmonic imaging has been proposed as a novel method for isolating ultrasound microbubble signals while suppressing the surrounding tissue signals. In the review article titled “Recent Experiences and Advances in Contrast-Enhanced Subharmonic Ultrasound,” the authors summarized recent advances in the use of subharmonic imaging in vivo. It is anticipated that this new technique may improve the signal-to-noise ratio compared with the current CEUS technique, which will finally provide more detailed information about the microcirculation in the ROI and it has more potential in the future.

The contributions in this special issue illustrate the recent advances in the area of CEUS. The results and the opinions have enriched the understanding of CEUS, which is helpful for clinical practice. It can be expected that CEUS may have a more extended application in the future.

*Hui-Xiong Xu* *Hans Peter Weskott* *Ji-Bin Liu* *Rong-Qin Zheng*
